# Public Preference for Electric Vehicle Incentive Policies in China: A Conjoint Analysis

**DOI:** 10.3390/ijerph17010318

**Published:** 2020-01-02

**Authors:** Wenbo Li, Ruyin Long, Hong Chen, Baoqi Dou, Feiyu Chen, Xiao Zheng, Zhengxia He

**Affiliations:** 1 Business School, Jiangsu Normal University, No.101 Shanghai Road, Xuzhou 221116, China; doubaoqi7@163.com (B.D.); 6020180084@jsnu.edu.cn (X.Z.); hezhengxia@163.com (Z.H.); 2 School of Management, China University of Mining and Technology, No.1 Daxue Road, Xuzhou 221116, China; hongchenxz@cumt.edu.cn (H.C.); chenfeiyu@cumt.edu.cn (F.C.)

**Keywords:** consumer preference, electric vehicle, incentive policies, conjoint analysis

## Abstract

In order to mitigate energy consumption and greenhouse gas emission in the transportation sector, countries around the world have generally adopted electric vehicles (EVs) as a new development direction of the automobile industry. Although the Chinese government has issued a series of incentive policies to promote EVs, the ownership of EVs is still insufficient due to low public purchasing enthusiasm. Thus, to better realize the promotion goal of EVs, public preference for EV incentive policies is worth investigating. Based on a large sample survey (N = 1039), this study investigated public preference for various incentive policies by using the conjoint analysis method. The results suggest that less than one third of consumers have a better understanding of the incentive policies, while more than half of the consumers know little about these policies. For consumers, the relative importance of different policy categories is ranked as follows: charging incentive policies, driving incentive policies, vehicle registering incentive policies, and purchasing incentive policies. As for different socio-demographic groups, consumers aged 26–30 years, with a monthly income higher than RMB 20,000, with high school, special secondary school, and masters (or above) educational levels regarded the relative importance of driving incentive policies as the highest; consumers from two-member families ranked purchasing incentive policies as the first one; consumers with a monthly income of RMB 15,001–20,000 and those from three-member families place registering incentive policies first; other consumers put charging incentive policies first. Based on the above results, this paper offers policy recommendations for improving consumer knowledge level of incentive policies as well as full consideration of their policy demands.

## 1. Introduction

With the improvement of the inhabitant living standard and large-scale urbanization, automobile ownership in China will continuously grow in the coming years. According to the prediction of the Ministry of Industry and Information Technology, the number of automobiles will surpass two hundred million by 2020. Existing studies show that tailpipe emissions, including carbon monoxide, hydrocarbons, and nitrogen oxides, have become the main source of air pollution in the metropolis (usually about 70%–80%) [1,2]. An obvious example is that hazy weather in China has become more and more frequent in recent years [3,4]. Thus, the tailpipe pollution caused by increasing automobiles cannot be neglected. In addition, China is the country with the second highest oil consumption in the world. Since domestic oil resources are insufficient and almost 70% of the consumed oil relies on imports into China, the rapid growth of the national economy has led to imbalances between oil demand and supply [5,6]. Without a doubt, the sharp increase in car ownership is a significant reason for the excessive dependence on oil imports. In the next few years, the oil demand will continue to increase with the rapid development of the automobile market, which will undoubtedly further aggravate the oil demand and supply imbalance.

In order to mitigate energy consumption and carbon emissions, the Chinese government has made great efforts to develop a low-carbon society [7,8,9,10]. The promotion of clean and energy-saving vehicles, especially electric vehicles (EVs), is an important aspect, which is also a breakthrough in the field of transportation reform worldwide [11]. Without an internal combustion engine, EVs do not exhaust tailpipe emissions and have almost zero pollution. The large-scale application of EVs can also reduce the dependence on fossil fuels as the electricity can be converted from green energy, such as water, solar, and wind power [12,13,14]. Even though the generation source is dominated by fossil fuels, EVs are still greener than conventional fuel vehicles (CFVs) because power plants have a higher energy conversion efficiency than internal combustion engines and the emissions caused by power plants are easier to clean than the millions of internal combustion engines on the road [15]. In addition, the concentrated emissions of power plants are generally far away from dense population areas, which causes less danger to public health [16]. In addition, the recent slow economic growth in China has resulted in a lower electricity consumption and higher electricity surplus, while the electricity generation capacity is still increasing. The large-scale application of EVs can consume and store some of the surplus electricity, which can help mitigate the electricity surplus problem during the peak period as well as reduce the pressure on the power grid. Thus, compared with CFVs, EVs are better, not only for environmental protection, but also for the reduction of the dependence on oil from other countries.

As an effective way to realize the energy conservation goal in the transport sectors, EVs were proposed as the direction of the automobile industry by the National Development and Reform Commission in the Automobile Industry Development Policy in 2004 [17]. Moreover, the decision of the State Council on Accelerating the Fostering and Development of Strategic Emerging Industries even identified EVs as one of the seven strategic emerging industries in China. However, at that time, EVs were inferior to CFVs in many aspects, such as the high purchasing price (more expensive than their gasoline-powered counterparts), short driving range (no more than 200 km), long refueling time (6–8 h), and insufficient charging stations, so EVs did not become the first choice for automobile customers [18]. Since 2009, in order to promote the industrialization of EVs, the government has gradually issued a series of incentive policies during the market introduction stage, which created a good policy environment [17]. With the support of the above policies, the production and sale volume of EVs are continuously increasing. In 2015, the production and sale volume of EVs were 152,172 and 146,719, an increase of 2.1 times and 2.3 times, respectively, compared with the same period of the previous year. It can be seen that the incentive policy was the first impetus during the initial introduction stage. The vigorous development of EVs cannot exist without the driving force provided by various incentive policies [19].

Although the development of EVs in China has achieved considerable progress, the market share of EVs is still low at present. In 2005, the market share was only 0.8%, and with a recent sharp increase in EVs, the market share reached 2.7% in 2017. These increasing values largely depend on the adoption of government and public institutions, but it is difficult to realize the large-scale application of EVs based on the present model. As private cars account for a large proportion of all automobiles, it is expected that the promotion of EVs will also depend on private consumers. The EV is a new automobile product, and consumers generally hold negative views and have doubts about its performance, stability, and convenience. For example, many customers still believe that the driving range of EVs is very short, while in fact, the driving range of present EVs can entirely satisfy their daily travelling demand [20]. Such cognitive barriers mean that customers do not easily accept EVs. At present, private consumers purchase less than one third of the total number of EVs sold in China, suggesting that only a few EVs are purchased by private consumers [21]. The government also issued a series of incentive policies that focus on satisfying private consumers, such as a purchase subsidy, tax exemption, parking benefits, and driving privileges, but the private EV market is still facing the dilemma of a “hot policy” and “cold market”. Since it has been difficult to make a great breakthrough in the EV performance and technology level in recent years, incentive policies are more likely to determine consumer acceptance of EVs compared with any other aspect. Therefore, there is an urgent need to improve existing policies to make EVs more attractive. In order to achieve this goal, it is essential to understand the policy demands of private consumers, thereby issuing more effective policies to reduce consumer doubt (e.g., issuing policies to ensure dedicated EV parking spaces to solve charging anxiety) and to lead to consumers accepting EVs more easily.

EVs are in the initial development stage, and present EV promotion faces a series of challenges due to the gradual elimination of government subsidies. The main purpose of this paper is to evaluate consumer preference of EV incentive policies, thereby fully understanding the existing defects and finding a future improvement direction from the micro-perspective. The findings can be used to provide an empirical basis to adjust upcoming incentive policies in order to ensure market competitiveness and to better realize large-scale application of EVs.

## 2. Literature Review

With the pressure of energy conservation and environmental protection, a large number of incentive policies have been issued to promote EVs. Existing studies have divided current policies into several categories. For example, Zhang et al. (2014) suggested that present incentive policies mainly focus on the charging infrastructure, financial subsidy, tax exemption, and R & D investment [22]. In the study of Yuan et al. (2015), five aspects of incentive policies were analyzed and summarized: industrial development, development plan, demonstration project, fiscal subsidy, and tax exemption [23]. The Annual Report on New Energy Vehicle Industry in China divided incentive policies into six categories: macroscopic policies, demonstration policies, preferential tax policies, science and technology innovation policies, industry management policies, and infrastructure policies. Some studies focused on reviewing and summarizing EV incentive policies with regards to a certain country, such as China, Japan, the United States, Germany, Lithuania, Norway, and Austria. There are also some studies that compared EV-related policies in different countries. Zhang et al. (2014) reviewed EV-related policies in America, Europe, Japan, and China regarding financial incentives, technical supports, and charging infrastructure, and they suggested that countries should learn from each other and that future policies could be combined with domestic socio-economic characteristics to attract more potential consumers [24]. Zhou et al. (2015) reviewed the incentive policies of EVs in the United States, China, Japan, and some European countries and found that the increasing EV market share was accompanied by the improvement of incentive policies [19].

As the large-scale application of EVs largely depends on the adoption of private consumers, many studies focused on the impact of incentive policies on individual adoption behavior. Currently, a large number of incentive policies have been proven to be the direct drivers of individual adoption [25,26,27,28], and Ko and Hahn (2013) further illustrated the significant positive effect of continuous policy improvement [29]. In addition, Zhang et al. (2013) put forward that incentive policies not only have a direct effect, but also indirect effects through the following four aspects: economic savings, performance attributes, environmental concern, and psychological demands [30]. Tang and Wu (2013) took the incentive policy, resident income, and oil price as independent variables and EV sales as the dependent variable and found that incentive policies had a significantly positive effect on EV sales based on a panel regression analysis [31]. Wang et al. (2017) divided incentive policies into three categories, that is, fiscal incentive policy, information provision policy, and convenient use policy, and found that the three policies had significant positive effects on EV adoption [32].

Among all the policies, economic incentive policies are generally believed to be the most effective. For example, Sierzchula et al. (2014) found that a financial subsidy, tax exemption, road toll exemption, and free registration had the greatest impact on EV adoption by analyzing consumers from 30 countries [33]. Aasness and Odeck (2015) proposed that the reason why Norway is becoming a global forerunner in the electromobility field lies in the economic incentive policies (e.g., tax reduction/exemption, road toll exemption, free parking, and driving privileges), which can help consumers save numerous expenses [34]. Bjerkan et al. (2016) found that more than 80% of respondents in Norway believe that reduced value-added tax and purchase tax are the key factors that motivated them to adopt EVs, which suggests that prepurchasing cost reduction is an effective measure [35]. Based on survey data, Ko and Hahn (2013) used a mixed logit model to evaluate the utility of major factors and found that the higher the amount of subsidies, the higher the utility of consumers, and that a one-off subsidy was more efficient than installments [29].

As for other policies, through analyzing owners of fuel-efficient cars in Stockholm, the capital of Sweden, Whitehead et al. (2014) found that the traffic congestion exemption policy could effectively improve the ownership of a fuel-efficient car, whereas the free parking policy is not as effective [36]. Bjerkan et al. (2016) suggested that road toll exemption and driving privileges only strongly affected specific groups [35]. Wang et al. (2017) suggested that driving privileges are more important than fiscal incentive and information provision policies [32]. By modeling questionnaire survey data, Wang et al. (2017) suggested that purchasing and driving restrictions played an important role in prompting EV adoption [37]. A charging fee reduction, driving privileges, and purchase tax exemption also had positive effects on purchase intention, but the effect of road toll exemption and free parking was not obvious. In another study, Wang et al. (2017) used macroscopic data from 2013 to 2014 to again verify the effectiveness of the traffic restriction policy, and they also found positive effects of the free registration and charging infrastructure construction [38]. Similarly, Ma et al. (2017) found a positive correlation between the EV market share and several incentive policies, including the purchasing subsidy, tax reduction/exemption, and purchasing and driving restrictions [39]. Some studies also found that current incentive policies are not as good as expected. For example, Hoen and Koetse (2014) proposed that incentive policies can indeed lead consumers to purchase EVs, but cannot eliminate public doubts about EV shortcomings [40]. Green et al. (2014) even stated that current incentive policies aim at realizing the large-scale application of EVs, so they focus on consumers of mainstream markets [41]. However, these policies cost too much and are not efficient, so it may be better to pay more attention to the niche market.

Overall, there are a number of studies trying to provide deeper insight into understanding the effect of EV-related incentive policies. However, public attitudes towards these policies, which largely decide policy implementation effects, are ignored. Not only are consumers the recipients of EV policies, their attitudes towards these policies also determine whether they are willing to adopt EVs. Presently, only a few studies have focused on public understanding of and preference for EV-related policies. Lane and Potter (2007) suggested that the government should take measures to let consumers fully understand incentive policies of energy-saving automobiles, otherwise it is difficult for these policies to work as expected [42]. Coad et al. (2009) used 1500 Swiss families as a survey sample and discussed the degree of public support for information provision and financial incentive policies [43]. The result showed that the effect of one policy mainly depended on the support degree of potential consumers. Similarly, Sovacool and Hirsh (2009) suggested that frequent changes to EV-related policies could result in consumers resisting EVs [44]. Consumers who enjoy relevant tax reduction and exemption policies may resist or even refuse to adopt EVs when these policies cannot save as many expenses as before. On the contrary, Caulfield et al. (2010) reported that based on a collection of tax policies in Ireland since 2008, public perceptions of these policies did not effectively influence the choices made [45]. Rezvani et al. (2015) suggested that more studies could further analyze public attitudes towards specific policies through various methods, which can provide more references for future policy-making [46]. Thus, in order to eliminate the present knowledge gap, this paper intends to use a conjoint analysis method to analyze consumer preference for EV incentive policies in China. The results can provide references for future policy-making to better realize the widespread application of EVs.

## 3. Methods

### 3.1. Survey Design

The questionnaire of this study mainly included three parts: public knowledge of EV incentive policies, public preference for EV incentive policies, and personal information. Public knowledge of EV incentive policies was collected by referring to relevant questions in the studies of Kang and Park (2011) [47], Zhang et al. (2013) [30], and Li et al. (2016) [17]. Public preference for EV incentive policies was evaluated based on a conjoint analysis method, and more details are given in the next paragraph. Since this study mainly focused on EV purchasing and driving incentive policies, we divided these policies into four categories by summarizing existing research results. These four categories are purchasing, registering, driving, and charging incentive policies. The first includes government subsidies, purchasing tax exemption, and insurance discount; the second refers to no purchasing restriction, registering priority, and a dedicated license plate; the third covers no driving restriction, parking discount, road toll exemption, and vehicle inspection priority; the final incorporates public charging infrastructure construction, a subsidy for constructing private charging piles, and a charging discount. In order to ensure the quality of the questionnaire, before the formal investigation, we conducted a small-scale survey of 100 respondents in Nanjing, which is the capital of Jiangsu Province and also one of the national EV pilot cities. According to the survey results and feedback from the respondents, the final questionnaire was obtained by revising the original version.

The first step was to investigate public knowledge of EV incentive policies. In order to ensure the accuracy and usefulness of the results, survey participants needed to clearly know what these policies refer to. Thus, before the survey began, the main content of each policy was introduced by trained investigators to participants. For example, the wording for the parking discount was as follows: “A parking discount indicates that EVs are not charged for parking at temporary parking spots on city roads and public parking lots in a short time (usually 2 h), and in social parking lots, the parking fee can still be given a discount.” Then, participants needed to answer the familiarity degree of this policy. A five-point Likert scale was used to reflect the degree of familiarity, and points 1–5 denote very unfamiliar, relatively unfamiliar, general, relatively familiar, and very familiar, respectively.

The second step was to evaluate public preference for EV incentive policies by using the conjoint analysis method. The main feature of this method is that individual purchase decisions can be observed under an imaginary situation [48]. In reality, when consumers are making purchase decisions (e.g., purchasing a private car), they consider all the attributes of different products (e.g., price, brand, appearance, and storage space) and carefully compare them. After consideration, the utility of each product is known by themselves. The conjoint analysis method was in accordance with the above decision-making process for the following steps. Firstly, attributes and attribute levels needed to ensure that target products were simulated; secondly, respondents had to evaluate the utility of a specific product within an imaginary context; and thirdly, a mathematical statistical method was used to analyze public preference of different attributes and attribute levels. If the main characteristics of the target product can be abstracted into several attributes, the conjoint analysis method is applicable. The main purpose of this study was to analyze public preference for different policies, so we needed to observe individual purchase decisions under different policy mixes that are made up of policies from different categories. Based on existing studies, the number of policies of a policy mix should be limited, generally to no more than 5 [49]. Too many attributes increase the answer difficulty and confuses respondents, thereby reducing the quality of data collection. On the contrary, insufficient attributes lead to information loss and low reliability of the results. According to this criterion, the defined four categories and their corresponding policies were set as attributes and attribute levels, respectively (see Table 1). We then used an orthogonal experimental design to mix them to form a series of representative survey tasks. Through the use of the orthogonal experimental design function of SPSS, we obtained 25 policy mixes (an example is shown in Table 2). Respondents needed to answer whether they would purchase an EV under each policy mix, and a five-point Likert scale was given to the respondents. Points 1–5 represent very unlikely to purchase, unlikely to purchase, uncertain, likely to purchase, and very likely to purchase, respectively.

The third part was to collect respondents’ personal information, including gender, age, marital status, education, monthly income, family population size, the number of family cars owned, the number of family EVs owned, housing type, type of location, charging conditions at home and nearby, and so on.

### 3.2. Data Collection

The formal investigation selected Beijing, Shanghai, Nanjing, Guangzhou, Hangzhou, and Hefei as target cities, and the reasons are as follows. Firstly, they are all national EV pilot cities, and their EV ownerships are the top in the country, so consumers in these cities have a greater chance to access EVs and know related incentive policies. Secondly, these cities are all provincial capitals, and their car ownership is at the forefront of other cities. Therefore, it is reasonable to forecast that there are more potential consumers who might be concerned about EV-related policies. The final goal of this paper is to provide evidence for policy makers to better understand consumer demand, thereby helping to promote EVs. Thus, potential car buyers are ideal survey respondents, and auto stores are places where one can find such respondents. Thus, the questionnaires were distributed in Auto 4S stores, and a number of trained investigators were invited to help us to collect questionnaires. The reasons we chose Auto 4S stores as the survey area are as follows. To begin with, consumers in Auto 4S stores are more likely to purchase cars or have a purchase intention, so they are more familiar with cars and EVs. Secondly, consumers in Auto 4S stores differ in terms of gender, age, and income, and we can obtain a sample of respondents with different demographic characteristics. Table 3 reflects the sample structure of this survey. Many of the respondents were male, young (31–35 years old), well-educated (bachelor’s degree or higher), had three or four family numbers, had one car, were in the middle-income group, and lived in urban areas. In the survey process, some small gifts, such as car key chains, deodorizing charcoal bags, and phone brackets, were provided as encouragement to increase response quality. Finally, 1039 valid questionnaires were received, and the questionnaire numbers for Beijing, Shanghai, Nanjing, Guangzhou, Hangzhou, and Hefei were 178, 183, 169, 173, 159, and 177, respectively.

### 3.3. Model Specification

In this section, individual preference for different policies was generated in the process of understanding EV incentive policy mixes. This preference influences both the individual evaluation of current incentive policies as well as the final purchase decision. If consumers do not know whether they really want to purchase an EV, they carefully compare existing incentive policies to make a final decision. Based on their comparisons, the conjoint analysis method was then used to analyze and evaluate their preference of EV incentive policies. This method is a multivariate statistical analysis in essence, and it is usually used to observe and evaluate individual psychological reactions. The core idea is that one product is made up of a series of attributes, and the utility that consumers can obtain is derived from these attributes. This method has been applied in many areas, such as transportation [48], medical devices [50], and communication equipment [51]. Thus, by setting an imaginary context that individuals are going to purchase an EV and regard different incentive policies as attributes, we could determine their preference for each incentive policy. To be specific, respondents’ preference for different policies was evaluated by their score of the incentive policy mixes. Through a conjoint analysis model, the utility value, which represents individual importance perception of each incentive policy, was estimated. The conjoint analysis model is shown in Equation (1):

(1) $$Y=c+\sum_{i=1}^{n}\sum_{j=1}^{m}a_{ij}x_{ij}$$

where *i* denotes the 1 to the *n* policy incentive category and *j* denotes that the *i* category has *m* incentive policies. When policy *j* of category *i* is in the policy mix, the value of *x_ij_*, which is a dummy variable, is 1; otherwise, it is 0. *a_ij_* denotes the utility value of policy *j* of category *i*. *c* is an intercept that denotes the utility when consumers do not choose any policy mixes. *Y* denotes the score of one policy mix. According to Equation (1), the utility value of each policy can be calculated. By using Equation (2), we can calculate *I_i_*, which is the relative importance of each incentive policy category. In this equation, *Max(a_ij_)* and *Min(a_ij_)* denote the highest and lowest utility value of the *i* policy incentive category.

(2) $$I_{i}=\left\{Max(a_{ij})-Min(a_{ij})\right\}$$

The relative importance of the *i* incentive policy category can then be calculated by Equation (3):

(3) $$W_{i}=I_{i}/\sum_{i=1}^{n}I_{i}$$

Through the above steps, we can obtain the relative importance of the four categories and the utility value of each policy. This study selected SPSS as the statistical software to analyze survey data based on the conjoint analysis module. After modeling the data, model reliability and validity, which are generally reflected by the respective Pearson and Kendall correlation coefficients, should be tested. The former coefficient reflects the model fitting degree, and its value should be higher than 0.8. The latter coefficient reflects the model internal validity, and its significance level should be less than 0.05, which suggests a good model predictive capability.

## 4. Results

Public knowledge of different EV incentive policies is shown in Table 4. The results of this section were obtained from a five-point Likert scale, so a higher value means that respondents had a better understanding of a policy. In this part, values below and above 3 were defined as inferior and superior values, respectively. Except for no purchase restriction, the inferior value of other incentive policies accounted for more than 50%, suggesting that more than half of the respondents were not familiar with them. The superiority value of all the incentive policies accounted for less than 30%, suggesting that less than one third of respondents were familiar with them. To be specific, the mean value of public knowledge was ranked in descending order as follows: no purchasing restriction (2.92), dedicated license plate (2.77), tax exemption (2.64), charging infrastructure construction (2.6), registering priority (2.6), charging discount (2.57), purchase subsidy (2.56), driving privilege (2.55), parking discount (2.44), subsidy of private charging piles (2.43), road toll exemption (2.37)/registering priority (2.37), and insurance discount (2.32). The mean value of the four policy categories was ranked as registering (2.76), charging (2.53), purchasing (2.51), and driving (2.43).

Table 5 summarizes the results of public preference for each incentive policy. Pearson’s correlation coefficient and Kendall’s correlation coefficient were 0.988 and 0.878, respectively, and their significance levels of the two-tailed test were both less than 0.05, suggesting that the fitting degree of the estimated model was good and the results accurately reflect the public policy preference. From the perspective of the incentive policy category, the charging category was of greatest public concern, and its relative importance was 30.132%. The relative importance of the driving, registering, and purchasing categories was 27.831%, 26.415%, and 22.176%, respectively. Moreover, public preference for incentive policies was ranked as tax exemption (0.192), purchase subsidy (0.145), road toll exemption (0.14), charging discount (0.137), subsidy of private charging piles (0.134), charging infrastructure construction (0.09), dedicated license plate (0.083), driving privilege (0.074), no purchasing restriction (0.068), registering priority (0.039), parking discount (0.036), vehicle inspection priority (0.022), and insurance discount (−0.063).

In order to further analyze public preference for EV incentive policies among different socio-demographic groups, the relative importance of the four policy categories was calculated, and the results can be seen in Table 6. With regard to most socio-demographic groups, charging incentive policies attracted greater concern, followed by driving, purchasing, and registering incentive policies. The difference lies in the following aspects: (1) respondents aged 26–30 years old ranked the relative importance of driving incentive policies as the first one; (2) respondents with high school, special secondary school, and masters (or above) educational levels regarded the relative importance of driving incentive policies as the highest; (3) respondents with a monthly income of RMB 15,001–20,000 and higher than RMB 20,000 valued registering and driving incentive policies, respectively; (4) respondents from two-member families paid more attention to purchasing incentive policies, while single respondents valued driving incentive policies; and (5) respondents from families with three or more cars regarded registering incentive policies as more important.

## 5. Discussion

### 5.1. Public Knowledge for EV Incentive Policies

Before analyzing public preference for electric vehicle incentive policies, public knowledge of each policy was discussed from the following four aspects.

Regarding the aspect of EV registering, it can be seen that the gap between public knowledge of no purchasing restriction and a dedicated license plate was not very large, while the public did not know much about the registering priority policy. One possible reason for this is that in car purchasing-restricted cities (e.g., Beijing and Shanghai), no purchase restriction of EVs benefits potential consumers, especially first-time car buyers. EV purchasers can avoid the license plate lottery and auction, and this advantage is always advertised by the automobile dealers [52]. Moreover, dedicated license plates of EVs began in 2015, and in the investigated cities, many EVs have already used these new license plates, which can often be noticed on the road. Therefore, consumers are familiar with this incentive policy.

Regarding the aspect of EV charging, consumers know much about the charging infrastructure construction policy, but pay less attention to the charging discount and subsidy of private charging piles. In 2009, China launched a national project named Ten Cities and One Thousand Vehicles, the main focus of which is the construction of public charging infrastructures [17]. With the increasing number of charging infrastructures, consumers are more familiar with relevant policies. However, private charging policies have only been issued in recent years, so consumers only have a limited understanding of them.

Regarding the aspect of EV purchasing, consumers know more about the tax discount, but are not fully aware of the purchase subsidy. The government has gradually canceled the purchase tax reduction of CFVs, but the purchase tax exemption of EVs is constantly implemented. Thus, the gap between these two policies has caused growing public concern. In addition, the subsidized price of EVs is generally advertised, and most consumers do not know the magnitude of government subsidies. Because the implementation of the insurance discount is recent and this policy can only help people save limited costs, consumers also do not know much about this incentive.

Regarding the aspect of EV driving, public knowledge of driving privilege is the highest. In China, the driving privilege mainly refers to no driving restrictions, which are of great importance to consumers living in large cities. People who drive EVs for daily travel are not limited by most driving restrictions, and such a convenience is known by the public.

### 5.2. Public Preference for EV Incentive Policies

A deep analysis of the public preference of electric vehicle incentive policies is presented as follows.

With respect to the charging policy category, respondents had the highest preference for the charging discount. The preference for the subsidy of private charging piles was the second highest, and its utility value was 0.134. The charging infrastructure construction policy had the smallest utility (0.09). At present, the cheap driving cost, which is closely related to the charging discount policy, attracts many consumers, so the preference for this policy is high. Moreover, charging at home is more convenient than using public charging infrastructures. Consumers are inclined to charge at home or nearby, and the subsidy of private charging piles can save installation costs for consumers [33].

With regard to the driving policy category, respondents had the highest preference for the road toll exemption policy, followed by the driving privilege, parking discount, and vehicle inspection priority. Currently, private CFVs are exempt from annual checking for 6 years, and owners can save time by making an appointment in advance. Thus, vehicle inspection priority of EVs has a limited attraction for consumers. According to the current parking discount policy, the government can only ensure that EV drivers enjoy parking benefits in public parking lots, and this is the reason why consumers do not have a high preference for the parking discount policy [11]. The reasons why respondents have a high preference for the road toll exemption and driving privilege policies lies in two aspects: one is helping car owners continuously save travel costs, and the other is guaranteeing that EVs are not affected by traffic control policies.

Within the registering policy category, the dedicated license plate policy has the highest public preference. By contrast, respondents have a lower preference for the no purchasing restriction policy, and their preference for vehicle inspection priority is the lowest. Presently, although EVs attract government subsidies, some other privileges, such as no driving restrictions and free parking, are always difficult to implement in reality [53]. Dedicated license plates can help traffic control departments better distinguish the identity of EVs, thereby ensuring that EV drivers enjoy those privileges [11]. According to the results of this survey, consumers indeed prefer the dedicated license plate policy. In addition, the license plate lottery and auction restrict the purchase and use of CFVs to a great extent. No purchasing restriction is the main force that promotes the growth of EV ownership, so consumers are more concerned about this policy.

Within the purchasing category, respondents’ preference for the tax discount was the highest, followed by the purchasing subsidy and insurance discount. In existing studies, a purchasing subsidy was believed to be the chief factor in EV adoption intention [37,39]. However, the results in this study show that consumers had a higher preference for the tax discount rather than the purchasing subsidy. The reason for this may lie in the high purchasing cost of EVs. After deducting government subsidies, the purchase price of most EVs is still higher than that of their gasoline counterparts. In addition, the government subsidy decreases year by year and is expected to be cancelled in 2020, so consumers generally have a low preference for the purchasing subsidy [11]. At present, CFV owners need to pay a certain amount of vehicle purchase tax as well as vehicle and vessel tax according to their prices and displacements, so the tax exemption of EVs attracts consumers to a certain extent. In addition, how much tax consumers should pay depends on the car purchase price. With regard to EVs, the purchase tax is calculated by the price before deducting the government subsidy, so the tax exemption policy can help consumers save numerous costs. The utility value of the insurance discount is negative, and the possible reason for this is that this discount aims to exempt compulsory insurance for vehicle traffic accident liability for new EVs in the first year and can only help consumers save a small expense (usually about RMB 1000). Compared with the results of existing studies, there are some new findings in this paper. Financial policies (e.g., purchasing subsidy and tax reduction policy) are regarded as the most effective ones [37,38,52]. However, the results of this paper show that these polices attracted the lowest public concern, and consumers believed that the registering and driving incentive policies were more important. On the contrary, most consumers regarded charging incentive policies as the most important, while this kind of policy has been proven to be less effective than financial policies in existing studies. It is suggested that a high purchasing cost is no longer the main concern of the public, who have begun to pay attention to convenience in daily driving. The future policy direction could be adjusted to focus on this change, and several suggestions will be discussed in the next paragraph.

### 5.3. Policy Implications

Based on the above results, the government can further improve two aspects of EV incentive policies. To begin with, in order to improve public knowledge of EV incentive policies, the government could provide more chances for the public. Moreover, since the purpose of launching various incentive policies is to promote the adoption of EVs, the government should also focus on public policy needs. Specifically, this can be achieved in the following ways.

First, continuous implementation of a purchase tax exemption could be ensured in order to establish public adoption confidence. With the reduction of purchase subsidies, purchase tax exemption has become a highlight that attracts consumers to choose EVs. Compared with CFVs, the price of EVs is relatively high, and a purchase tax exemption can help consumers reduce purchase costs. At present, the vehicle purchase tax is 8.547% of the selling price (including tax). For example, consumers who purchase an EV, the price of which is about RMB 200,000, can receive an RMB 17,000 tax exemption. According to the results of this study, consumers prefer the purchase tax exemption policy. Previous studies also showed that the implementation of this policy can really prompt consumers to purchase EVs. In the future, the government could consider extending the implementation period of this policy and use this advantage to attract more consumers. In addition, the government could timely update the vehicle catalogue of the purchase tax exemption to ensure that more EVs can be chosen by consumers.

Second, the EV-dedicated license plate could be actively promoted, since the implementation of this policy has a positive guiding effect for potential consumers. At present, the number of EVs in China is more than 1 million, but this number is still far lower than that of CFVs. When consumers need to purchase a private vehicle, EVs are still not their first choice. With the increasing number of dedicated license plates on the road, these bold green license plates look like mobile advertisements and can be seen as a symbol of social identity. Through these license plates, EV drivers and users can transmit information such as “energy conservation”, “emissions reduction”, and “social responsibility” to the people around them, which plays a positive role in establishing their self-confidence and honor. Therefore, the government could actively promote dedicated license plates of EVs in order to display their publicity function; in addition, the government needs to upgrade the traffic control system to accurately identify dedicated license plates, thereby ensuring various privileges of EVs.

Third, the related policies of charging infrastructures should be improved. Current incentive policies pay more attention to public charging infrastructures instead of their private counterparts. Therefore, more investment could be put into the construction of private charging equipment. This view has also been verified by Skippon and Garwood (2011), and their results showed that consumers are willing to pay appropriate expenses to install and upgrade self-charging piles at home [54]. Specifically, the government could provide certain financial supports for consumers who need to construct self-charging piles and strengthen cooperation among automobile, financial capital, and power grid enterprises to create a better environment for private charging pile construction. As for public charging infrastructures, policies that encourage stakeholders (e.g., shopping malls, hotels, and pedestrian streets, and gas stations) to actively invest in the construction of charging infrastructures could be issued.

## 6. Conclusions

To gain a better understanding of public policy demand and promote EV uptake, public knowledge of and preference for EV incentive policies were analyzed by a descriptive statistical analysis and a conjoint analysis method, respectively. The results showed that less than one third of consumers are familiar with EV incentive policies, whereas more than half of them are unfamiliar with these policies. For consumers, the relative importance of different policy categories is ranked as follows: charging incentive policies, driving incentive policies, vehicle registering incentive policies, and purchasing incentive policies. As for different socio-demographic groups, consumers aged 26–30 years, with a monthly income higher than RMB 20,000 with high school, special secondary school, and masters (or above) educational levels regarded the relative importance of driving incentive policies as the highest; consumers from two-member families ranked purchasing incentive policies as the first one; consumers with a monthly income of RMB 15,001–20,000 and those from three-member families place registering incentive policies first; other consumers put charging incentive policies first. Based on the above results, some implications were put forward to further improve EV promotion strategies as well as provide a reference for the formulation of EV incentive policies in the future. The limitations of this paper are as follows. First, the surveyed cities are mainly distributed in eastern coastal and central areas of China, which is insufficiently representative of the whole country and fails to include consumers in the western and northeastern regions. In addition, respondents with certain demographic characteristics are insufficient, such as participants aged older than 60 years. The following studies can further expand the survey cities to northeastern and midwestern China, ensuring higher representativeness of respondents. Moreover, the survey areas do not need to be limited to auto stores, and more respondents from driving schools, car rental companies, and similar areas can be selected. Secondly, with the promotion of EVs in China, the surveyed cities are no longer limited to national pilot cities, and more representative cities can be included to improve the universality of the results. Thirdly, the results only illustrate the utility value of each policy, whereas the relationship between each policy and the likelihood of purchasing is not mentioned. Future studies could collect sufficient data and fully develop a model to analyze this issue.

## Figures and Tables

**Table 1:** Attributes and attribute levels.

Attributes	Attribute Levels
Purchasing Incentive Policies	Purchasing subsidy; Tax exemption; Insurance discount
Registering Incentive Policies	No purchasing restriction; Registering priority; Dedicated license plate
Driving Incentive Policies	Driving privilege; Parking discount; Road toll exemption; Vehicle inspection priority
Charging Incentive Policies	Charging infrastructure construction, Subsidy of private charging piles; Charging discount

**Table 2:** An example of policy mixes.

Enjoyment of the Following Incentive Policies Determines Whether You Will Purchase an EV:	
Purchasing:	Purchase subsidy
Registering:	Dedicated license plate
Driving:	No purchasing restriction
Charging:	Charging discount
□Very unlikely to purchase □Unlikely to purchase □Uncertain □Likely to purchase □Very likely to purchase	

**Table 3:** Demographic characteristics of respondents.

Demographics		Percentage
Gender	Male	47.83%
	Female	52.17%
Age (years)	25 and younger	9.01%
	26–30	26.36%
	31–40	30.39%
	41–50	22.01%
	51–60	11.41%
	Older than 60	0.82%
Education	Junior high school or below	3.26%
	High school/special secondary school	7.88%
	Junior college	19.02%
	Bachelors	41.85%
	Masters or above	27.99%
Family Population	1	4.35%
	2	8.97%
	3	49.46%
	4	22.83%
	5 or more	14.4%
Family Car Number	0	33.15%
	1	55.98%
	2	10.05%
	3 or more	0.82%
Monthly Income (RMB)	Less than 2000	15.22%
	2001–4000	20.11%
	4001–6000	26.36%
	6001–8000	15.22%
	8001–10,000	8.97%
	10,001–15,000	7.34%
	15,001–20,000	4.62%
	20,001–30,000	1.63%
	More than 30,000	0.54%
Location	Urban	73.37%
	Suburban	19.57%
	Rural	7.07%

**Table 4:** Public knowledge of electric vehicle (EV) incentive policies.

Incentive Policies		Mean Value	Standard Deviation	Inferior Value	Superior Value
Purchasing (2.51)	Purchase subsidy	2.56	1.347	50.7%	23.7%
	Tax exemption	2.64	1.388	50.7%	28.6%
	Insurance discount	2.32	1.360	61.5%	22.6%
Registering (2.76)	No purchasing restriction	2.92	1.502	41.9%	40.6%
	Dedicated license plate	2.77	1.540	49.6%	37%
	Registering priority	2.6	1.404	51.2%	28.9%
Driving (2.43)	Driving privilege	2.55	1.417	52.3%	28.1%
	Parking discount	2.44	1.378	57.7%	25%
	Road toll exemption	2.37	1.392	61%	25.3%
	Vehicle inspection priority	2.37	1.354	59.2%	22.6%
Charging	Charging discount	2.57	1.42	52.6%	28.6%
	Subsidy of private charging piles	2.43	1.359	57.8%	25.1%
	Charging infrastructure construction	2.6	1.4	51.7%	29.4%

**Table 5:** Public preference for EV incentive policies.

Policy Category	Relative Importance	Incentive Policies	Utility Value
Purchasing	22.176%	Purchase subsidy	0.145
		Tax exemption	0.192
		Insurance discount	−0.063
Registering	26.415%	No purchasing restriction	0.068
		Dedicated license plate	0.083
		Registering priority	0.039
Driving	27.831%	Driving privilege	0.074
		Parking discount	0.036
		Road toll exemption	0.140
		Vehicle inspection priority	0.022
Charging	30.132%	Charging discount	0.137
		Subsidy of private charging piles	0.134
		Charging infrastructure construction	0.090
Pearson’s R = 0.988		Sig. = 0.000	
Kendall’s tau = 0.878		Sig. = 0.000	

**Table 6:** Relative importance of EV incentive policies among different socio-demographic groups.

Socio-Demographic Characteristics		Policy Category			
		Purchasing	Registering	Driving	Charging
**Gender**	Male	22.859%	19.862%	28.182%	28.327%
	Female	21.499%	19.096%	27.483%	31.922%
Age (years)	18–25	22.377%	19.079%	28.351%	30.194%
	26–30	27.328%	19.644%	28.681%	23.128%
	31–40	20.609%	18.769%	26.916%	33.707%
	41–50	27.297%	20.603%	21.39%	30.71%
	51–60	20.782%	19.362%	28.283%	31.574%
Education	Junior high school or below	20.596%	26.813%	17.468%	35.124%
	High school/special secondary school	19.278%	20.959%	32.289%	27.474%
	Junior college	21.493%	20.039%	26.625%	31.842%
	Bachelors	22.131%	19.385%	26.821%	30.793%
	Masters or above	22.968%	19.032%	29.427%	28.573%
Monthly Income	Less than RMB 2000	20.199%	18.632%	29.987%	31.182%
	RMB 2001–4000	27.99%	19.744%	22.783%	29.483%
	RMB 4001–6000	22.256%	19.047%	28.056%	30.641%
	RMB 6001–8000	27.793%	19.531%	22.954%	29.722%
	RMB 8001–10,000	21.767%	18.715%	28.631%	30.888%
	RMB 10,001–15,000	20.48%	20.42%	24.31%	28.905%
	RMB 15,001–20,000	27.984%	31.212%	18.742%	22.062%
	More than RMB 20,000	29.53%	17.029%	30.974%	22.467%
Family Population	1	21.157%	21.087%	29.948%	27.808%
	2	29.363%	17.466%	21.808%	27.016%
	3	21.757%	19.995%	28.091%	30.157%
	4	21.192%	19.801%	28.162%	30.846%
	5 or more	28.206%	17.76%	22.306%	31.729%
Family Car Number	0	22.892%	20.322%	27.812%	28.974%
	1	21.789%	19.088%	27.72%	30.722%
	2	21.983%	18.378%	29.242%	30.398%
	3 or more	22.559%	31.886%	13.872%	31.684%
Location	Urban	21.861%	19.763%	27.58%	30.252%
	Suburban	22.518%	19.046%	28.249%	30.187%
	Rural	23.785%	18.271%	28.813%	29.132%
